# The Association Between Glycemic Variability and Myocardial Infarction: A Review and Meta-Analysis of Prospective Studies and Randomized Trials

**DOI:** 10.7759/cureus.11556

**Published:** 2020-11-18

**Authors:** Zinab Alatawi, Hyder Mirghani

**Affiliations:** 1 Family Medicine, University of Tabuk, College of Medicine, Tabuk, SAU; 2 Internal Medicine, University of Tabuk, Tabuk, SAU

**Keywords:** glucose variability, myocardial infarction, hba1c variability

## Abstract

Diabetes mellitus is a major risk factor for vascular complications and mortality, glycemic variability (GV) has emerged as a measure of time and magnitude of plasma glucose, its association with cardiovascular complications is controversial. The current study aimed to assess the association of GV with myocardial infarction. An electronic literature search was conducted in PubMed, Medline, and Google Scholar databases for relevant articles. Articles in the English language during the period from 2010 to April 2020 were eligible. The keywords fasting blood glucose variability (FBGV), glucose variability, myocardial infarction, and acute coronary syndrome, were used with the protean OR and AND. Out of the 185 articles retrieved, only seven full texts fulfilled the inclusion and exclusion criteria. The author's name, year of publication, the study type, number of patients, and the results were extracted. There were seven full texts, one from Brazil, one from Australia, two from the USA, and three from Asia. Two were randomized controlled trials and five were prospective cohorts (included 109,058 participants). A significant negative association was found between GV and myocardial infarction, odd ratio (OD) 1.93, 95% CI=1.08-3.44, P-value=0.03, I^2 ^for heterogeneity=87%, P-value=0.0001. However, it is difficult to reach a conclusion due to the small number of the included studies and the high heterogeneity observed. Further well-controlled trials using the same methods are needed to resolve the issue.

## Introduction and background

Glycemic variability (GV) is a major issue when evaluating the degree of glycemic control. It also correlates with hypoglycemia, and GV increases from prediabetes to advanced type 2 diabetes (T2D) and is still high in type 1 diabetes mellitus. Many metrics exist to measure GV (%Coefficient of Variation and the standard deviation are the most popular) [1].

Glucose fluctuation is associated with inflammation, oxidative stress, and endothelial dysfunction, factors that are to blame in the pathogenesis of vascular damage [2]. Increased variability of fasting blood glucose (FBG) is independently associated with the development of T2D [3,4]. There is an increasing awareness regarding the possible detrimental effects of GV on the coronary arteries and the role of targeting it as an interventional strategy for prevention [5]. Retrospective studies [4,6,7] showed that glycemic variability is linked to myocardial infarction, stroke, and all-cause mortality among prediabetes and normoglycemic individuals. The literature on the association of fasting blood glucose variability (FBGV) with coronary artery disease scares. The current review aimed to assess the relationship between FBGV and myocardial infarction. 

## Review

Methodology

Eligibility Criteria According to PICOS (Patient/Population, Intervention, Comparison and Outcomes)

Type of studies included: All randomized controlled studies and prospective on humans were included, retrospective cohorts, case reports, case series, animal, and experimental studies were excluded. Participants: All adults in whom myocardial infarction and GV were measured. Outcomes measures: Studies were included if they assessed glucose variability and myocardial infarction.

Information Sources and Search Methods

A systematic manual search was conducted in PubMed, Medline, and the first 100 articles in Google Scholar in the last 10 years from 2010 to April 2020; the following keywords were used, fasting plasma glucose variability, myocardial infarction, hemoglobin A1c (HbA1c) variability, and glycemia variability with protean OR, and AND in different combinations.

Study Selection and Data Extraction

**Figure 1:**
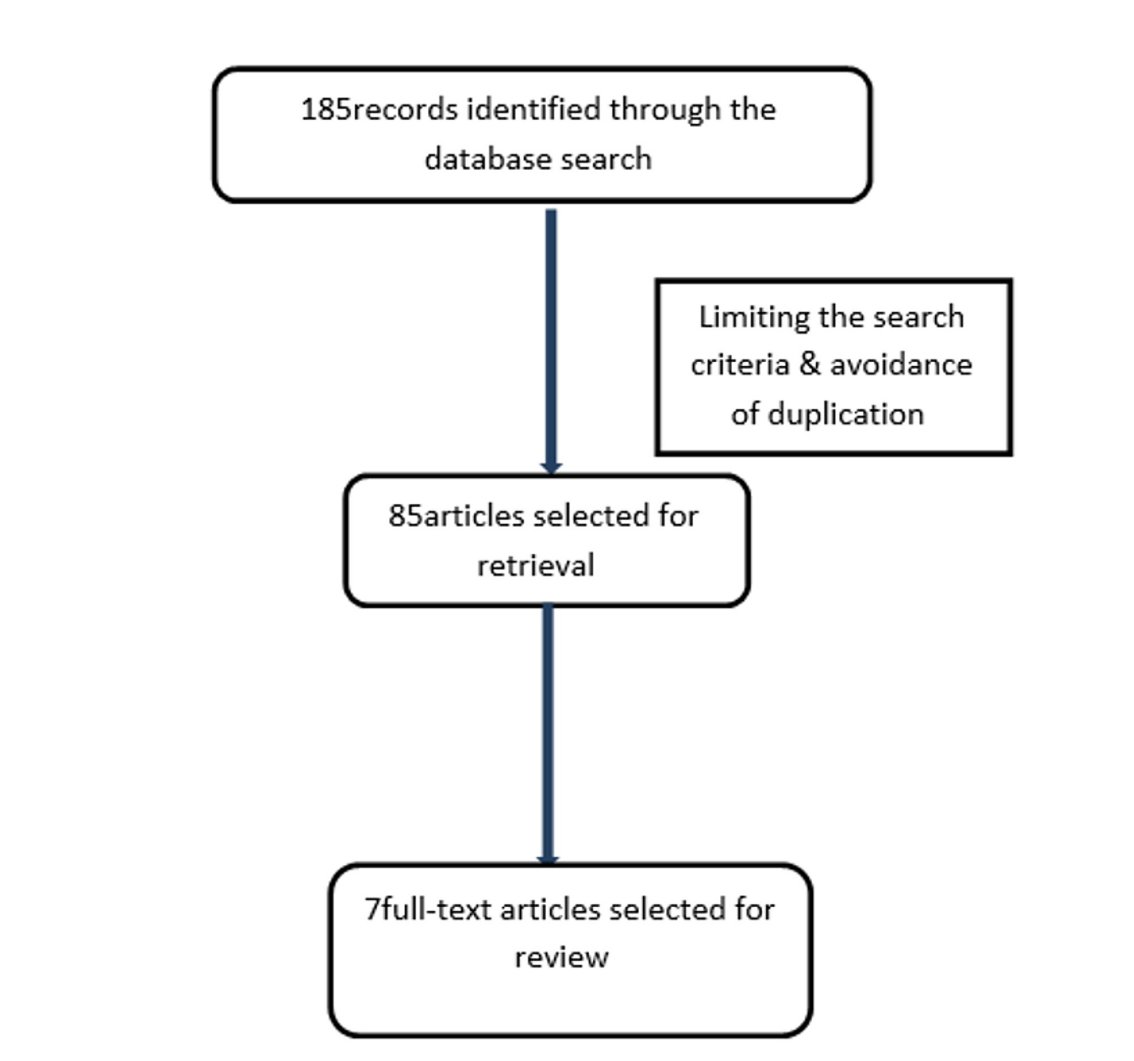
The different phases of the literature search

Two authors screened the titles and abstracts independently for relevant articles and irrelevant articles were removed, and the discrepancy between the two authors was solved by consensus. The author's name, year of publication, type of study, the number of patients included, and the findings of the studies were recorded. Out of the 185 articles screened, only seven full texts were eligible after applying the inclusion and exclusion criteria (Figure 1).

Statistical Analysis

RevMan 5.4 software (Cochrane, London, UK) was used for the meta-analysis. For glycemic variability (binary) risk ratios (RRs) with 95% confidence intervals (CIs) were combined across relevant studies, the random effects module was applied due to substantial heterogeneity (A P value ≤ 0.10 for Cochran’s Q test or an I2 ≥ 50% was suggestive). A two-tailed P < 0 05 was considered statistically significant for all analyses except heterogeneity tests.

Results

**Figure 2:**
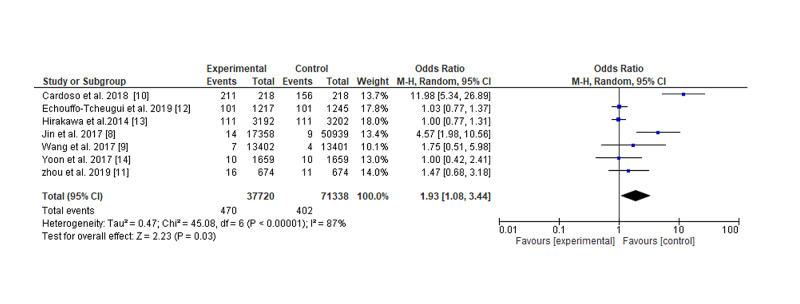
Glycemic variability and myocardial infarction risk

**Figure 3:**
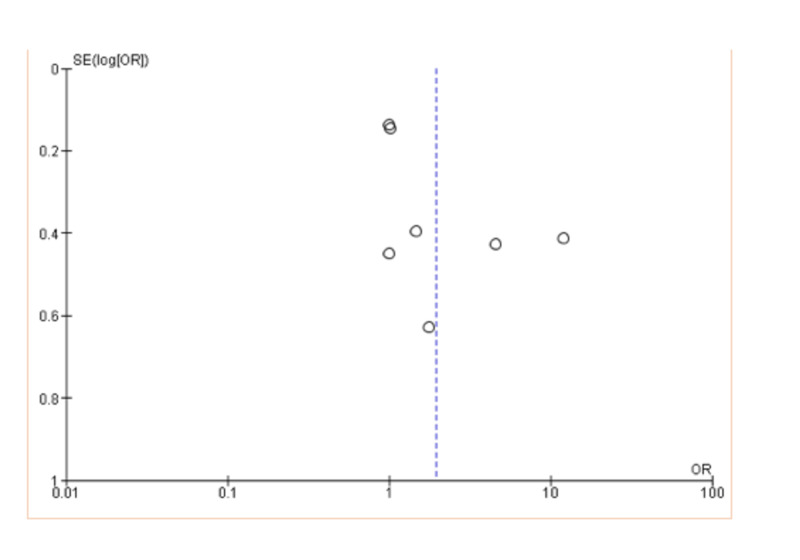
Glycemic variability and myocardial infarction risk SE: standard error, OR: odds ratio

There were seven studies, one from Brazil, one from Australia, two from the USA, and the rest from Asia. Two were randomized controlled trials and five prospective cohorts (Table 1). In the present review, of the seven studies included in the meta-analysis four showed a negative association between myocardial infarction and GV [8-11], while three showed a neutral effect [12-14], the studies included 109,058 participants. A negative significant association was found between glycemic variability and myocardial infarction, odd ratio, 1.93, 95% CI=1.08-3.44, P-value=0.03, I2 for heterogeneity=87%, P-value=0.0001 (Figures 2, 3).

**Table 1:** Fasting blood glucose variability association with cardiovascular risks and diabetes mellitus development RCT: randomized controlled trial, FPG: fasting plasma glucose test, DM: diabetes mellitus, FBG: fasting blood glucose, MI: myocardial infarction, T2D: type 2 diabetes, TIA: transient ischemic attack, CV: cardiovascular

Author	Year	Country	Type of study	No of patients	result
Hirakawa et al. [13]	2014	Australia	RCT	4,399 patients followed for 24 months	Glucose variability was associated with vascular damage and death
Jin et al. [8]	2017	China	Prospective	68,297 followed for 4 years	An increasing rate of FBG predicted future risk of MI.
Wang et al. [9]	2017	China	Longitudinal cohort	53 607 followed for 4.93 years	PG variability linked to mortality and cardiovascular disease irrespective of FPG
Yoon et al. [14]	2017	Korea	Prospective cohort	674 TIA and stroke (3 months)	The initial glycemic variability might increase CV events in acute ischemic stroke patients with diabetes
Cardoso et al. [10]	2018	Brazil	A prospective cohort	654 patients followed for 9.3 years	Glucose variability predicted both micro and macrovascular complications
Echouffo-Tcheugui et al. [12]	2019	USA	Prospective cohort	4982 followed for five years	visit-to-visit variability in FPG was associated with increased mortality
Zhou et al. [11]	2019	USA	RCT	1791 followed for 84 months	Fasting glucose variability was linked to mortality after controlling for hypoglycemia and lifestyle

Discussion

In the present review, of the seven studies included in the meta-analysis four showed a negative association between myocardial infarction and GV [8-11], while three showed a neutral effect [12-14], the studies included 109,058 participants. A significant negative association was found between GV and myocardial infarction: odd ratio, 1.93, 95% CI=1.08-3.44, P-value=0.03, I2 for heterogeneity=87%, P-value=0.0001. Smith-Palmer and colleagues [15] conducted a systematic review among patients with diabetes mellitus and found an association between glucose variability and myocardial infarction in type 1 diabetes, while the findings were inconsistent in type 2 diabetes, a review published in China [16] stated that GV may be a marker of increased progression of coronary disease and plaque vulnerability in contradiction to our finding; a plausible explanation might be the small number and different methods of the studies included, glucose variability is thought to induce more oxidative stress predisposing to vascular damage [17,18]. Although there is no gold standard measure for glucose variability, standard deviation estimated from self-monitoring blood glucose and the mean amplitude of glycemic excursions calculated from the continuous glucose monitoring are in common use.

The current study is unique in that it is the first to conduct a meta-analysis and include randomized and prospective study, the limitations were the small number of the included studies and the heterogeneity reported that might be due to the different methods of assessing glucose variability. Nevertheless, the current finding brought an issue that needs consideration and calls for further studies in this important area of research that was not well-covered in the current literature. Further well-controlled studies and including continuous glucose monitoring, strict dietary approach, and using the standard deviation to assess both intraday and inter-day variability are needed.

## Conclusions

Glucose variability is negatively associated with the development of myocardial infarction. However, it is difficult to reach a conclusion due to the small number of the included studies and the high heterogeneity observed. 
